# A Novel method for quantifying fluctuations in wearable derived daily cardiovascular parameters across the menstrual cycle

**DOI:** 10.1038/s41746-024-01394-0

**Published:** 2024-12-23

**Authors:** Summer R. Jasinski, David M. Presby, Gregory J. Grosicki, Emily R. Capodilupo, Victoria H. Lee

**Affiliations:** 1 Whoop Inc., Data Science and Research, Boston, MA USA; 2 Department of Computational Biology, University of Lausanne, Lausanne, Switzerland; 3 Swiss Institute of Bioinformatics, Lausanne, Switzerland

**Keywords:** Biomarkers, Reproductive biology, Cardiovascular genetics

## Abstract

Currently, knowledge of changes in cardiovascular function across the menstrual cycle and how these changes may inform upon underlying health is limited. Utilizing wrist-worn biometric data we developed a novel measure to quantify and investigate the cardiovascular fluctuation (i.e. cardiovascular amplitude) within resting heart rate (RHR) and heart rate variability (RMSSD) across 11,590 participants and 45,811 menstrual cycles. Within participants, RHR and RMSSD fluctuated in a regular pattern throughout the menstrual cycle, with population RHR_min_ and RMSSD_max_ at cycle day 5, RHR_max_ at day 26, and RMSSD_min_ at day 27. Cardiovascular amplitude was attenuated (*p* < 0.05) in older participants and participants using birth control, suggesting the novel metric may mirror differences in hormonal fluctuations in these cohorts. Longitudinal tracking of cardiovascular amplitude may offer accessible non-invasive monitoring of female physiology and underlying health across the menstrual cycle.

## Introduction

In postmenarcheal, premenopausal females, disruptions to the menstrual cycle outside of pregnancy may indicate an underlying health concern, and it has been suggested that the menstrual cycle be used as a vital sign^1^. For instance, missed periods (i.e., secondary amenorrhea) are often related to low estrogen and progesterone levels, and low progesterone can result in premenstrual dysphoric disorder^2,3^. Additionally, those experiencing irregular menstrual cycles have been found to have a higher risk of coronary heart disease, cancers, and osteoporosis later in life^4–6^. Unfortunately, recognizing these cycle disruptions can be challenging as it may take several months before an individual identifies irregular or missed menses. A practitioner may also need to follow up with invasive blood tests to properly diagnose the underlying cause, further delaying the time until treatment. However, developing strategies that assist in rapidly identifying menstrual irregularities and facilitating earlier treatment should improve patient outcomes.

Menstrual cycle tracking via smartphone applications has become increasingly widespread, with over 200 million downloads of menstruation or fertility tracking applications globally^7^. Simultaneously, wearable technology has been consistently identified as one of the top fitness trends for several consecutive years^8–10^, with approximately one in three U.S. adults using a wearable health monitor in 2023^11^. Since wearable technology provides continuous and accurate cardiovascular measures such as resting heart rate (RHR) and the root mean square of successive differences measure of heart rate variability (RMSSD), and these cardiovascular measures are known to vary across the menstrual cycle^4–7^, the combination of wearable technology and menstrual cycle tracking offers an intriguing opportunity to monitor health across the reproductive lifespan and to rapidly identify menstrual irregularities. Relatedly, recent research suggests that wearables can be used for non-invasive and automatic tracking of fertility and pregnancy status^12,13^. Thus, wearable technology presents an attractive lens to evaluate female physiology across the menstrual cycle, and a unique opportunity to explore how individual factors such as age, body mass index (BMI), and birth control status may influence cycle-related physiological fluctuations.

The present study examined the magnitude of variation in RHR and RMSSD across the menstrual cycle in a large, free-living cohort using a wrist-worn device with motion and photoplethysmography (PPG) capabilities. To investigate the significance and degree of cardiovascular fluctuations across the menstrual cycle, we derived a novel “cardiovascular amplitude” metric, which we subsequently leveraged to explore the influence of individual factors on fluctuations in cardiovascular metrics. We hypothesized that cardiovascular function would fluctuate in a regular and predictable pattern across the menstrual cycle, and that age, BMI, and hormonal birth control would influence the magnitude of cardiovascular amplitude. If validated, monitoring of cardiovascular dynamics via wearable devices could serve as an easily accessible and objective indicator of female physiology with the potential to improve our ability to track cycle health beyond standard journal-based methods.

## Results

**Fig. 1:**
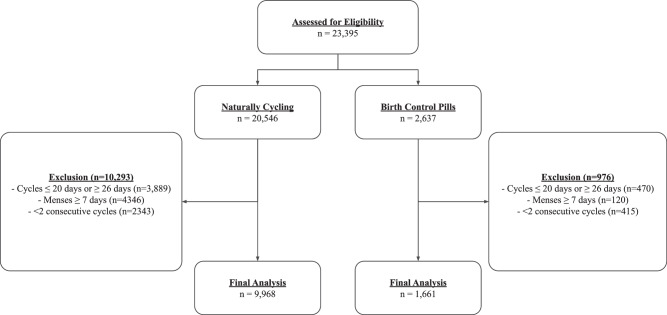
Diagram of included participants. Diagram of participant exclusion criteria illustrating the number of participants excluding at each step of data refinement. Two pathways are shown illustrating the naturally cycling cohort on the left side and the birth control pills cohort on the right side. In total 23,395 participants were assessed for eligibility; 9968 participants were included in the naturally cycling cohort and 1661 participants were included in the birth control pill cohort.

A total of 11,590 participants met the inclusion criteria for the study, as illustrated in Fig. 1. In total 9968 individuals were included in the naturally cycling cohort and 1661 in the birth control pill cohort. Data was collected from global participants, with the majority of data collected from the United States (69.9%), Great Britain (8.0%), Australia (4.2%), Germany (4.2%), Ireland (1.4%), the Netherlands (1.3%), the UAE (1.1%) and Switzerland (1.0%), with 92 other countries accounting for the remaining 8.9% of participants.

Eligible participants recorded 1,241,929 days of data and 45,811 unique menstrual cycles during the study period. Participant demographics and cardiovascular and menstrual cycle overview data are provided in Table 1. The mean duration of all menstrual cycles was 27.42 days (±2.16 days), with menstrual bleeding recorded on average for 4.66 days (±1.05 days).

**Table 1:** Participant Demographics

Attribute	All participants	Naturally cycling	Hormonal birth control pills
Age	34.88 yrs. (±7.32)	35.53 yrs. (±7.23)	30.88 yrs. (±6.60)
BMI	24.56 kg/m^2^ (±4.32)	24.61 kg/m^2^ (±4.35)	24.29 kg/m^2^ (±4.09)
Resting heart rate (RHR)	59.69 BPM ( ± 7.55)	59.54 BPM ( ± 7.53)	60.55 BPM ( ± 7.62)
Heart rate variability – (RMSSD)	60.41 ms (±27.17)	59.93 ms (±27.05)	63.46 ms (±27.79)
Day kilojoules	7,529.69 kJ (±1,004.13)	7,529.20 kJ (±1,015.42)	7,532.84 kJ (±928.46)
Menstrual cycle length	27.42 days (±2.16)	27.37 days (±2.25)	27.72 days (±1.44)
Menstrual bleeding length	4.66 days (±1.05)	4.71 days (±1.04)	4.36 days (±1.09)
Days of data available	107.16 days (±79.01)	107.30 days (±79.64)	103.77 days (±72.75)

Overview of cohort demographics for all participants, naturally cycling participants (primary analysis), and participants on hormonal birth control pills (secondary analysis). The mean (±SD) of each value is reported.

### Population model

**Fig. 2:**
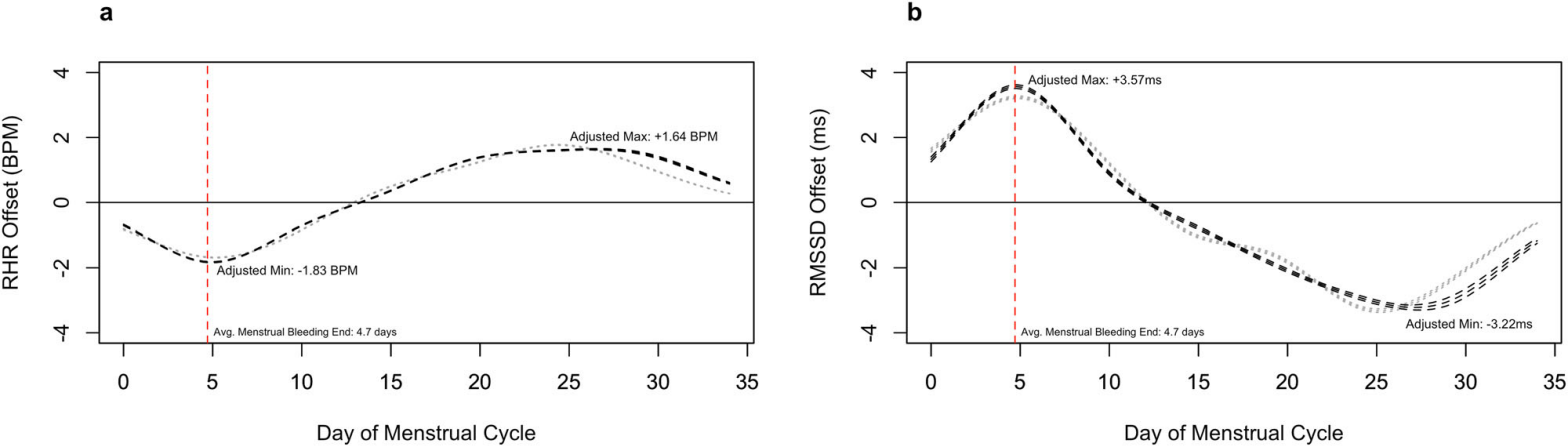
Population-Level GAMM of RMSSD HRV offset and RHR offset during the menstrual cycle. The partial dependence plot of the day of the menstrual cycle (D) from the population-level GAMM modeling the offset from the cycle-mean RHR and RMSSD in the naturally cycling cohort. Adjusted models are represented by the black dashed line, unadjusted models by the grey dotted line. The) models for RHR offset values are displayed in (**a**), models for RMSSD offset are displayed in (**b**). A red dashed line indicates the mean end of menstrual bleeding. Boundary lines indicate the 95% CI for estimates.

Analysis of the population-level GAMM of naturally cycling participants revealed a significant relation (*p* < 0.001) between the day of the menstrual cycle and metric offset from the cycle mean for both RHR (Fig. 2a) and RMSSD (Fig. 2b) in both unadjusted and adjusted models, confirming a robust association between menstrual cycle day and these cardiovascular metrics. RHR offset from cycle mean decreased during the beginning of each menstrual cycle, reaching a nadir nearest to day 5 (day 4.81, RHR offset: −1.83 BPM), after which RHR rose to its highest point at approximately day 26 (day 26.44, RHR offset +1.64 BPM) and maintained a positive offset through the conclusion of the menstrual cycle. The dynamics of the RMSSD offset as a function of the day of the menstrual cycle followed an inverse pattern to RHR with a maxima nearest to day 5 (day 4.81, RMSSD offset + 3.57 ms) and a nadir nearest day 27 (day 27.13, RMSSD offset −3.22 ms). All additional variables included in the adjusted model defined were significant (*p* < 0.05; Table 2).

**Table 2:** Model Statistics for RHR and RMSSD model, Naturally Cycling

	Naturally Cycling: Unadjusted RHR				Naturally Cycling: Adjusted RHR					Naturally Cycling: Unadjusted RMSSD				Naturally Cycling: Adjusted RMSSD			
*Predictors*	*Estimates*	*CI*	*Statistic*	*p*	*Estimates*	*CI*	*Statistic*	*p*	*Predictors*	*Estimates*	*CI*	*Statistic*	*p*	*Estimates*	*CI*	*Statistic*	*p*
Intercept	0.00	−0.01 – 0.01	0.00	1.000	−0.43	−0.47 – −0.40	−22.05	<0.001	Intercept	0.00	−0.02 – 0.02	0.00	0.997	1.10	0.96 – 1.23	15.99	<0.001
D			35018.24	<0.001			23685.13	<0.001	D			9534.44	<0.001			6374.61	<0.001
pID			0.00	0.995			1.84	0.025	pID			0.00	0.955			1.96	0.022
Weekend (True)					0.90	0.89 – 0.92	121.83	<0.001	Weekend (True)					−1.77	−1.82 – −1.72	−69.03	<0.001
BMI					0.01	0.01– 0.01	9.25	<0.001	BMI					−0.02	−0.03 – −0.02	−8.78	<0.001
age							10.51	<0.001	age							4.11	0.006
kJ								<0.001	kJ							1667.51	<0.001
Observations	1064953				10284737				Observations	1069351				1032269			
R^2^	0.116				0.135				R^2^	0.034				0.043			

Estimates, Confidence Intervals (CI), test statistics, and *p*-values for the predictors in the adjusted (*n* = 1,028,437) and unadjusted (*n* = 1,064,953) population GAMM models of RHR offset as well as the adjusted (*n* = 1,032,269) and unadjusted (*n* = 1,069,351) population GAMM models of RMSSD offset. For the RHR (Unadjusted) and RHR (Adjusted) output, the model illustrates the relationship between D, pID, weekend, BMI, age, and kJ on the offset of RHR throughout the menstrual cycle in naturally cycling individuals. For the RMSSD (Unadjusted) and RMSSD (Adjusted) output, the model illustrates the relationship between D, pID, weekend, BMI, age, and kJ on the offset of RMSSD throughout the menstrual cycle in naturally cycling individuals.

### Individual cardiovascular amplitude measures

As established above, the minimum expected point in the population model for RHR offset occurred at approximately day 5 of the menstrual cycle, and the maximum occurred at approximately day 26. As day 26 is within 3 days of the end of the menstrual cycle, the mean of the final 7 days of each cycle was used to provide equivalent data in each cycle. Therefore, the cycle cardiovascular amplitude for RHR was defined as the mean value on days 2–8 (7-day mean centered on day 5) subtracted from the mean value of the final 7 days, and RHR_amp_ was defined as each participant’s mean cycle cardiovascular amplitude across all eligible cycles. The average RHR_amp_ was 2.73 BPM, and 93.6% of participants demonstrated a positive RHR_amp_ value. The cycle amplitude measure for RMSSD was defined as the mean value on days 2–8 (7-day mean centered on day 5) less the mean value from the final 7 days. The average RMSSD_amp_ was 4.65 ms, and 80.6% of participants demonstrated a positive RMSSD_amp_ value.

### Association of Age and BMI with Cardiovascular Amplitude

**Fig. 3:**
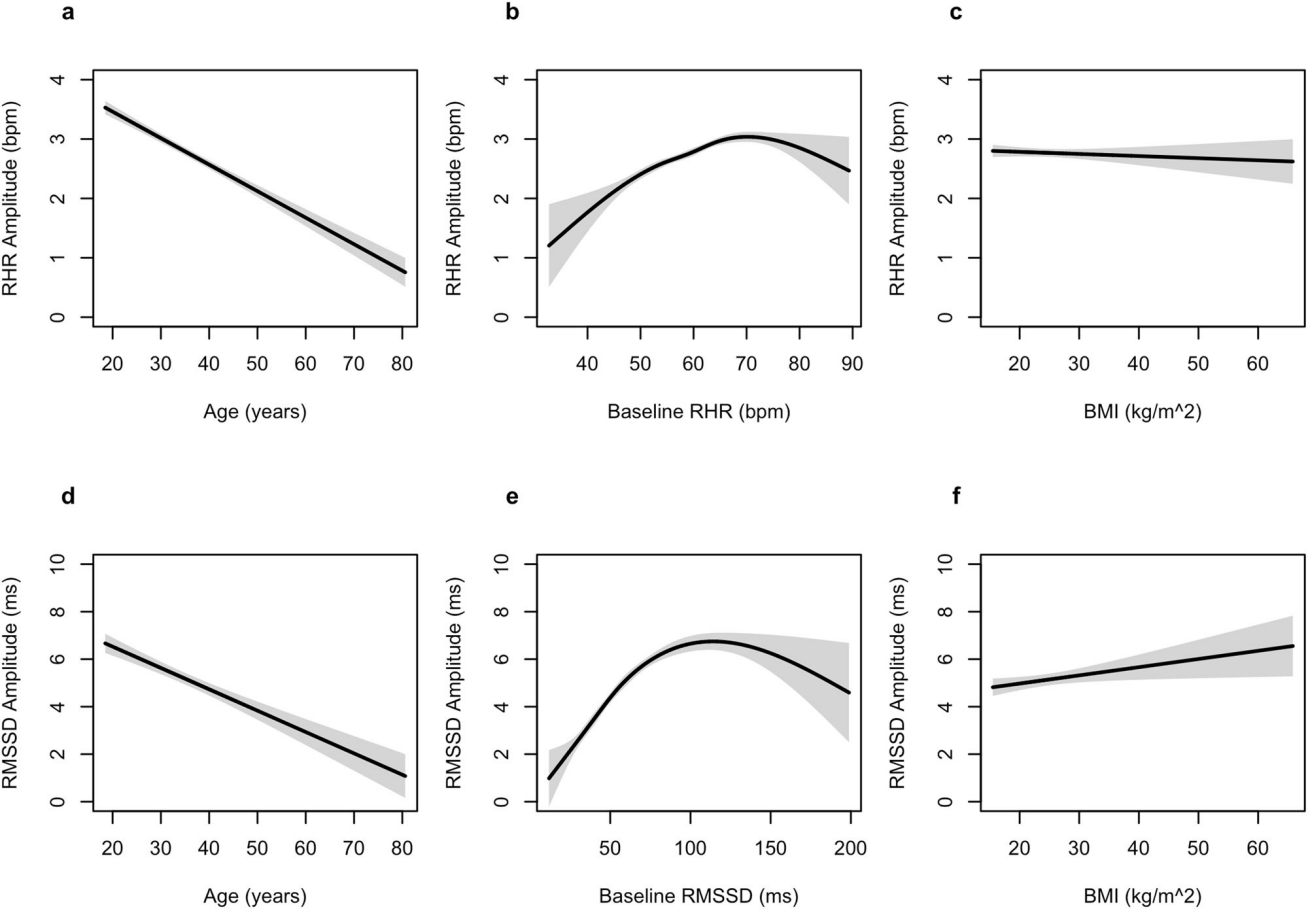
Results of GLM for RHR_amp_ and RMSSD_amp_. The partial dependence of age is plotted for (**a**) RHR_amp_ and (**d**) RMSSD_amp_ and the partial dependence of the four spline terms of baseline RHR is plotted for RHR_amp_ (**b**) and the four spline terms of baseline RMSSD is plotted for RMSSD_amp_ (**e**). The partial dependence of BMI is plotted for (**c**) RHR_amp_ and (**f**) RMSSD_amp_. Boundary lines indicate the 95% confidence interval for estimations.

The results from the GLMs suggest a negative association between age and RHR_amp_ (*β* = *−*0.04 95%CI[−0.05– −0.04], *p* < 0.001 and RMSSD_amp_ (*β* = − 0.09 95%CI[− 0.11 – −0.07], *p* < 0.001 (Fig. 3**;** Table 3). Notably, while the individual’s baseline value of the given metric (i.e. RHR or RMSSD) was a significant factor in the cardiovascular amplitude, the relationship was not linear. Rather than a linear relationship between cardiovascular amplitude and the individual’s baseline metric, RHR_amp_ and RMSSD_amp_ peak when baseline values are at the 50th–75th percentile (RHR_amp_ knot 3: *β* = *2*.76 95%CI[1.28− 4.24], *p* < 0.001, RMSSD_amp_ knot 3: *β* = 7.04 95%CI[4.35–9.73], *p* < 0.001). BMI in this analysis was not significantly associated with RHR, but there was a statistically significant association between BMI and RMSSD_amp_ (*β* = 0.03 95%CI[0.00–0.06], *p* = *0.026*).

**Table 3:** GLM Model Results

	GLM: RHRamp model			GLM: RMSSDamp model		
*Predictors*	*Estimates*	*CI*	*p*	*Estimates*	*CI*	*p*
Age	− 0.04***	− 0.05–− 0.04	<0.001	− 0.09***	− 0.11–− 0.07	<0.001
RHR ( < 54.3 BPM)	1.54***	0.88− 2.20	<0.001			
RHR (54.3 BPM - 59.3 BPM)	1.57***	1.12− 2.01	<0.001			
RHR (59.3 BPM - 64.4 BPM)	2.76***	1.28− 4.24	<0.001			
RHR ( > 64.4 BPM)	0.65*	0.03− 1.26	0.040			
BMI	0.00	− 0.01− 0.01	0.433	0.03*	0.00–0.06	0.026
RMSSD ( < 40.4 ms)				3.96***	2.83–5.08	<0.001
RMSSD (40.4 ms - 54.1 ms)				6.20***	5.07− 7.32	<0.001
RMSSD (54.1 ms - 73.3 ms)				7.04***	4.35− 9.73	<0.001
RMSSD ( > 73.3 ms)				2.63*	0.50− 4.76	0.016
Observations	9968			9968		
R^2^	0.040			0.062		

*p < 0.05, **p < 0.01, ***p < 0.001.

Estimates, Confidence Intervals (CI), test statistics, and *p*-values for the predictors in the RHR_amp_ (*n* = 9968) and RMSSD_amp_ (*n* = 9968) GLM models. This model illustrates the relationship between age, BMI, and Baseline RHR or RMSSD on cardiovascular amplitude throughout the menstrual cycle in naturally cycling individuals.

### Birth control cohort comparison

**Fig. 4:**
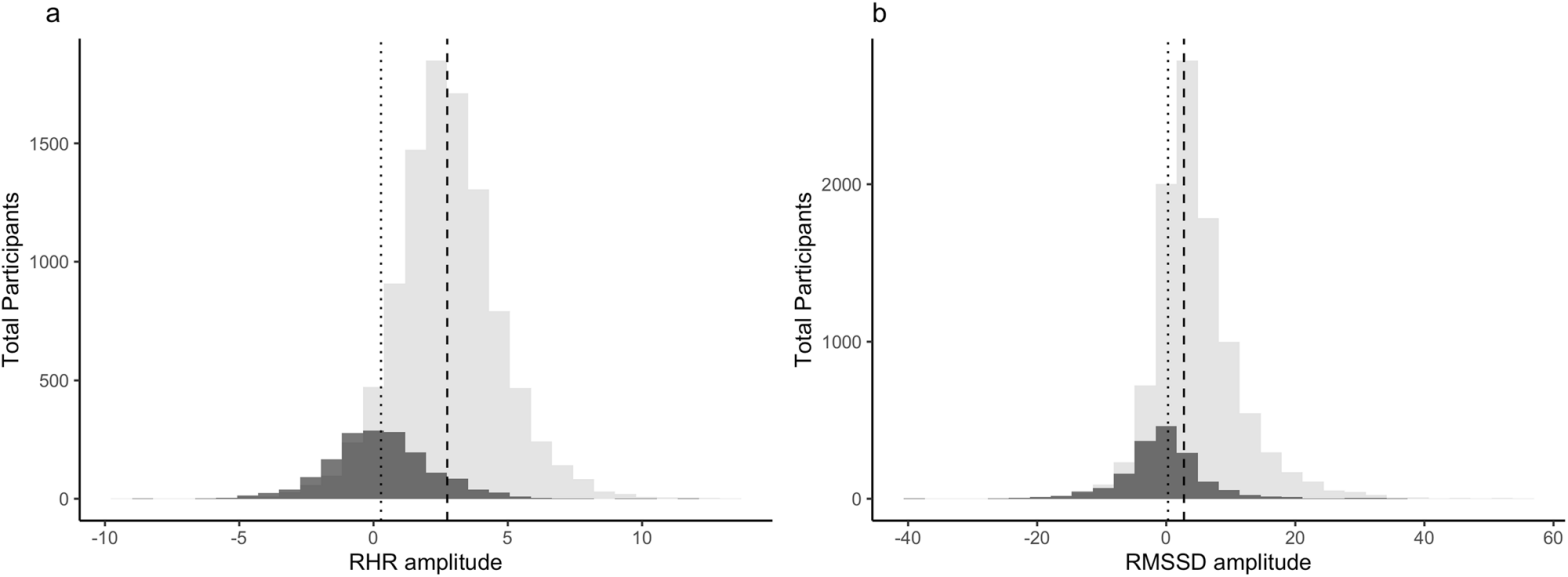
Distribution of novel cardiovascular amplitude metric for participants using a birth control pill and naturally cycling participants. Histogram plots for the distributions of RHRamp values are shown in (**a**) and the distributions of RMSSDamp values are shown in (**b**). In both (**a**) and (**b**), the naturally cycling cohort is displayed in light gray with the cohort mean represented by the dashed (---) line, while the birth control pill cohort is displayed in dark gray with the cohort mean represented by the dotted (…) line.

To investigate the relationship between birth control usage and cardiovascular amplitude, a sub analysis calculating individual cardiovascular amplitude was carried out in a cohort of confirmed birth control pill users (*n* = 1661), a population model of the birth control cohort is included in Supplementary Note 1. For RHR_amp,_ the mean value was 2.73 bpm (±1.95) for those naturally cycling and 0.28 bpm (±1.94) for participants using birth control pills. For RMSSD_amp,_ the mean value was 4.65 ms (±6.90) for those naturally cycling and −0.51 ms (±6.70) for participants using birth control pills (Table 4). There is a statistically significant difference between the two cohorts in the mean values of both RHR_amp_ (*p* < 0.001) and RMSSD_amp_ (*p* < 0.001) (Fig. 4). The results remained consistent in a small (*n* = 3322) age-matched subset of both cohorts (RHR_amp_ (*p* < 0.001) and RMSSD_amp_ (*p* < 0.001). Results for the age-matched subset are included in Supplementary Note 2.

**Table 4:** Cardiovascular Amplitude and Metric Offset Values for Naturally Cycling and Birth Control Cohorts

	RHR		RMSSD	
Mean (±s.d.)	Naturally cycling	Birth control pills	Naturally cycling	Birth control pills
Metric offset days 2–5	− 1.36 bpm (±1.07)*	0.03 bpm (±1.12)*	2.47 ms (±3.84)*	− 0.69 ms (±3.97)*
Metric offset final 7 days	1.37 bpm (±1.09)*	0.31 bpm (±1.04)*	− 2.18 ms (±3.78)*	− 0.18 ms (±3.55)*
Cardiovascular amplitude	2.3 bpm (±1.95)*	0.28 bpm (±1.94)*	4.65 ms (±6.90)*	− 0.51 ms (±6.7)*

*p < 0.001.

Mean values (±s.d) for the offset of the underlying metric of RHR and RMSSD for days two through five of the menstrual cycle, the final seven days of the menstrual cycle, and the difference between the two (cardiovascular amplitude) for both Naturally Cycling and Birth Control cohorts. Values are compared for statistical significance in the difference between the Naturally Cycling and Birth Control Cohorts using a two-sample t-test.

## Discussion

The purpose of this study was to examine the magnitude of the fluctuations in RHR and RMSSD across the menstrual cycle, which led to the derivation of a novel metric based on the cardiovascular amplitude of RHR and RMSSD. Secondarily, we sought to determine whether individual factors known to influence cycle-related variability in reproductive hormones (i.e., age, BMI, and birth control) may also be associated with variability in cardiovascular function. Our primary findings are that cardiovascular amplitude fluctuated in a regular and predictable pattern across the menstrual cycle and that fluctuations for RHR and RMSSD were tempered by increased age, hormonal birth control use, and increased BMI.

Consistent with our hypothesis, RHR and RMSSD fluctuated substantially and with consistent patterns across the menstrual cycle, both at the population and individual levels. More specifically, in this large cohort of naturally cycling and regularly menstruating females, the lowest RHR and highest RMSSD values were observed at the end of menstruation (i.e. days 3–7), and the highest RHR and lowest RMSSD values were observed in the late luteal phase and conclusion of the menstrual cycle (i.e. days 25–27). This pattern of menstrual cycle-related fluctuation in RHR and RMSSD metrics is comparable to previous studies from our group^14^, and others^15,16^, and is likely explained by cycle-related fluctuations in reproductive hormones^15^. Notably, there is an expanding market for objective, home-based cycle tracking methods such as urine and saliva testing to measure hormone levels. Unfortunately, these tests can be costly, burdensome, and are not always accurate^17,18^. Our findings in a large cohort of free-living individuals add to the existing literature in highlighting the possible value of considering wearable-derived cardiovascular metrics as an easily accessible and reliable indicator of female reproductive hormones across the menstrual cycle.

We observed significantly lower RHR_amp_ and RMSSD_amp_ in females reporting birth control pill use compared to those naturally cycling. Notably, the typical offsets from baseline during menstruation were relatively similar for participants naturally cycling and taking birth control but diverged significantly during the second half of the menstrual cycle (i.e., late luteal phase). We hypothesize that the significant fluctuations in cardiovascular parameters in naturally cycling participants may be due to progesterone increases in the late luteal phase, which are dampened in individuals on hormonal birth control^19^. This observation is consistent with previous findings from our group in females using combined oral contraceptives^14^. Taken together, these findings demonstrate significant attenuation of menstrual cycle-related cardiovascular variation by birth control, and highlight a major difference in cardiovascular physiology across the menstrual cycle in females who are naturally cycling compared with birth control users.

Reduced cardiovascular amplitude was also observed in females with higher body mass index and of older age. This reduction in cardiovascular amplitude in older females is consistent with findings demonstrating a gradual decline in anti-müllerian hormone, progesterone, and estrogen (i.e., reproductive hormones) among females throughout their reproductive life and approaching their last menstrual period^14,20,21^. With respect to body mass, individuals with abnormal BMI at either extrema are more likely to have anovulatory cycles, irregular menses, and hormonal imbalances^22^. Research has shown that premenopausal females with an elevated BMI exhibit reduced levels of estrogen and anti- müllerian hormone^22,23^. It is possible that the attenuated RMSSD_amp_ and RHR_amp_ in participants with abnormal BMI is connected to these underlying differences in hormonal health. Further research is warranted to better understand the association between the novel cardiovascular amplitude values derived in this study and direct measures of hormonal health.

Prior research exploring RMSSD across the menstrual cycle is limited. However, the High Frequency power (HF) frequency measure of HRV, which is strongly correlated with RMSSD and parasympathetic function, also exhibits a decrease in power during the mid-luteal phase of the menstrual cycle^24^. HF power levels are at their highest during the high-estrogen follicular phase, and at their lowest during the high-progesterone mid-luteal phase, however, whether these changes are ascribed to levels of estrogen, progesterone, or, more likely, a combination of each, is debated^25–27^. Examining the link between parasympathetic activity and menstrual cycle conditions investigations of individuals with premenstrual syndrome (PMS) and premenstrual dysphoric disorder (PMDD) found stronger phase to phase differences within the HF component of HRV were found in those with severe PMS, while a reduction in overall parasympathetic activity was observed in those with PMDD^27^. Autonomic Nervous System dysfunction has also been proposed as a driving factor in female fertility disorders due to a lack of sympathetic shift in the luteal phase^28^. This connection to menstrual cycle related disorders and fertility warrants further investigation into the change in RMSSD and RMSSD_amp_ in regards to the diagnosis and detection of PMS, PMDD, infertility, or other conditions.

Our findings should be interpreted in the context of our study’s strengths and limitations. First, we restricted our sample to include only regularly menstruating females. Thus, the generalizability of our findings to females with irregular menstrual cycles is unclear. Second, we relied on self-reported data concerning menstruation status and birth control methods, which can be subject to human error. Third, we did not have access to relevant sociocultural variables such as race/ethnicity and socioeconomic status that may influence reproductive physiology and cardiovascular function^29^. Additionally, our study would have benefited from direct hormonal measurements as well as a more thorough characterization of our cohort, inclusive of information regarding potential reproductive and/or cardiovascular health conditions as well as more detailed information regarding the use of hormonal contraception (e.g., duration of use, dose, brand, etc.), all of which may influence cycle-related physiology^24^. These weaknesses are balanced by the size of our dataset: over a million days of data and nearly 50,000 cycles from more than 10,000 regularly menstruating females, making our analysis among the largest to appear in the scientific literature to date. Additionally, our data collection featured state-of-the-art wearable technology that has been validated against gold-standard electrocardiogram and polysomnography measures and has been found to have a low degree of bias and low precision errors^30,31^.

In conclusion, we present cardiovascular amplitude as a novel method for quantifying the magnitude of cardiovascular fluctuations across the menstrual cycle in a large cohort of free-living menstruating individuals. Cardiovascular amplitude measures were tempered by increasing age, BMI, and birth control use. These findings add to a small but growing body of literature in support of changes in cardiovascular function across the menstrual cycle that mirror and may be causally related to fluctuations in reproductive hormones. Though further research is needed, the novel cardiovascular amplitude metric, derived through wearable technology, demonstrates an accessible and objective indicator of changes in female physiology within and across menstrual cycles, with the potential to inform upon underlying health.

## Methods

### Data collection

Daily data used for analysis were collected from study participants between January 2, 2022, and October 2, 2023, using a wrist-worn wearable device, the WHOOP Strap (Whoop, Inc., versions 3.0 or 4.0; Boston, MA, USA), which continuously measures vital signs, including RHR and RMSSD, RHR and RMSSD were calculated by the WHOOP cloud-based analytics platform during non-wake periods of the primary sleep episode^24^. The WHOOP cardiovascular and sleep measures have been validated in a general population against gold-standard electrocardiogram and polysomnography measures with a low degree of bias and low precision errors^30,31^. Respondent demographics including birthday, gender, height, and weight were available via study participants’ existing WHOOP profiles and were used to calculate participants’ age and BMI. Participants reported data regarding birth control type, birth control start date, and birth control stop date – if the use was discontinued – via an on-demand survey in the WHOOP app; data regarding menstruation status were self-reported daily via a survey in the WHOOP mobile application. Since data were not identifiable and were stored on a secure server, this study was determined to be exempt according to 45 CFR 46.104(d) by Salus IRB (https://www.versiticlinicaltrials.org/salusirb) and a full waiver of Informed Consent was granted as data collection involved minimal risk.

### Inclusion criteria

Participants who self-identified as female, were aged 18 or older at the time of data collection, had an active WHOOP membership, had previously reported use (or non-use) of hormonal birth control, and manually logged menstruation within the WHOOP mobile application were eligible for inclusion. In accordance with established guidelines for menstrual cycle research^32^, participants that reported menstrual cycles of irregular length (i.e., less than 21 or greater than 35 days)^33^, menstrual bleeding longer than 7 days^34^, or had fewer than 95% days of wear across a minimum of 2 consecutive menstrual cycles were excluded from the analysis. Given the known influence of hormonal birth control on hormone levels and cardiovascular dynamics, the primary analysis was restricted to participants reporting no regular birth control use or the exclusive use of barrier methods of birth control (i.e. naturally cycling) at the time of data collection^14,35^. A secondary analysis compared key results within naturally cycling participants with results for participants who reported the use of hormonal-based birth control pills. Participants could contribute data to both cohorts if they experienced a change in birth control status during the data collection period. All study participants consented to the use of their data for purposes of scientific research at the time of enrollment and could withdraw consent at any time.

### Data processing

RHR and RMSSD were normalized to within-user and within-cycle values by calculating the offset in absolute units from the cycle mean value with outliers removed. Outliers were defined as values above quartile 3 + 1.5*interquartile range (IQR) or below quartile 1–1.5*IQR for a given user and cycle. Following established guidelines, a menstrual cycle was defined as the time between the first self-reported menstrual bleeding and the onset of the next self-reported menstrual bleeding following a time of no reported menstrual bleeding^36^.

### Statistical analysis

To assess population-level trends in RHR and RMSSD across the menstrual cycle, a generalized additive mixed effect model (GAMM) was constructed for each metric using the “gam” package in R^37^. The variables examined for inclusion in the model as independent variables were the number of days since the onset of the menstrual cycle (*D*), the subject’s age in years (*age*), the subject’s daily kilojoule expenditure (*kJ*) from the prior day, whether the date was a Saturday or Sunday (*weekend*)^38^, BMI in kg/m^2^ (*BMI*), and a unique participant identifier (*pID)*. The following equations give the unadjusted and adjusted models for the applied GAMM:

**UNADJUSTED**

1 $${y}_{i}={f}_{1}(D)+{f}_{2}\left({pID}\right)+\varepsilon$$

**ADJUSTED**

2 $$\begin{array}{l}{y}_{i}={f}_{1}(D)+{f}_{2}\left({pID}\right)+{f}_{3}({age})\\\qquad+{f}_{4}({kJ})\,+{weekend}+{BMI}+\varepsilon\end{array}$$

Where *y*_*i*_ represents the offset from cycle mean of the metric (RMSSD or RHR), *f*_*1*_*(D)*, *f*_*3*_*(age)*, and *f*_*4*_*(kJ)* are smooth, non-linear functions of the previously defined variables *D, age*, and *kJ* respectively, and *f*_*2*_*(pID)* is the within-participant random effect associated with the variable *pID*.

**Fig. 5:**
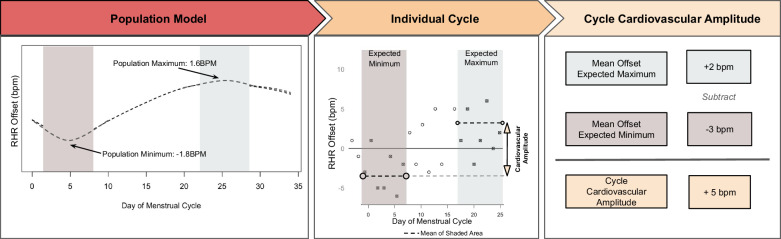
Visual methodology. Visual exploration of the assignment of individual menstrual cycle measures of cardiovascular amplitude. The ‘Population Model’ frame illustrates the population GAMM trend of Resting Heart Rate offset across the menstrual cycle. The population minimum and maximum are identified, and the surrounding days are shaded, with the grey shaded region encompassing the range of the expected minimum values and the blue shaded range encompassing the range of the expected maximum values. The second frame, ‘Individual Cycle’ illustrates the application to a single menstrual cycle. The individual resting heart rate offset values are represented by open circles. The dashed lines in each shaded region correspond to the mean values of resting heart rate offsets in the grey and blue shaded regions respectively. In the final pane, ‘Cycle Cardiovascular Amplitude’, the values from the prior panel are subtracted from one another, with the mean offset of the expected maximum minus the mean offset of the expected minimum representing the final cardiovascular amplitude metric.

The partial dependence plots for the number of days since the onset of the menstrual cycle (*D*) from the population GAMM models were used to determine the day within a standard menstrual cycle in which an individual’s RHR or RMSSD could be expected to reach a peak or nadir. The observed population peak and nadir were determined by the nearest day to the maxima and minima of the partial dependence plot in the population-level GAMM, respectively. A cycle amplitude for each participant-cycle was calculated as the difference between the 7-day mean centered on the expected peak and the 7-day mean centered on the expected nadir (Fig. 5). For instance, if the population model suggested a maximum on day 4 and a minimum on day 14, the cycle cardiovascular amplitude value for each participant-cycle was determined by subtracting the mean of days 11–17 from the mean of days 1–7. To establish individual cardiovascular amplitude, the mean of the cycle amplitudes from all of the participants’ eligible menstrual cycles was calculated. A 7-day mean was used to mitigate the impact of day-of-week effects^24^. If the maxima or minima were fewer than 3 days from the beginning or end of the cycle, the 7-day period was shifted to ensure a 7-day window of data was available for each subject. The derived participant cardiovascular amplitude values are denoted as RMSSD_amp_ and RHR_amp_ for RMSSD cardiovascular amplitude and RHR cardiovascular amplitude values, respectively.

To understand the relationship between known mediators of hormonal health and the novel cardiovascular amplitude metric, generalized linear models (GLM) were fit with the participant’s age (*age*), underlying cardiovascular metric value (*RHR and RMSSD respectively*), and BMI (*BMI)* as covariates to estimate values of RHR_amp_ and RMSSD_amp_. Natural spline functions with four knots were selected to model RHR and RMSSD data, while age and BMI were modeled linearly. The general formulae of the GAMM for RHR_amp_ (Eq. 3) and RMSSD_amp_ (Eq. 4) can be expressed as follows:

3 $${{RHR}}_{{amp}}={\beta }_{0}+{\beta }_{1}* {age}+{\beta }_{2}* {ns}({RHR},4)+{\beta }_{3}* {BMI}+\varepsilon$$

4 $${{RMSSD}}_{{amp}}={\beta }_{0}+{\beta }_{1}\,* {age}+{\beta }_{2}* {ns}({RMSSD},4)+{\beta }_{3}* {BMI}+\varepsilon$$

The cardiovascular amplitude metrics were also calculated for a cohort of participants who reported the use of birth control pills. The cardiovascular amplitude values for this cohort were calculated using the days for the expected minima and maxima derived from the naturally cycling population model. The cardiovascular amplitudes of naturally cycling participants were then compared to the cardiovascular amplitudes of the cohort of participants using hormonal birth control pills. In a supplementary analysis, the two cohorts were matched age using the ‘MatchIt’ package in R to create comparable populations (Supplementary Note 2)^39^. All data were expressed as mean ± standard deviation (SD) and differences were tested by a two-tailed *t*-test unless otherwise reported. All analyses were carried out in either Python (version 3.8) or R (version 4.2.2)^40^. Significance was set a priori at 0.05. The visualizations of the model outputs were generated using the sjPlot package^41^.

## Supplementary information

Supplementary File

## Data Availability

The data that support the findings of this study are not publicly available due to intellectual property concerns of WHOOP, Inc. Data may be made available upon request to the company via research@whoop.com for researchers who meet the criteria for access to confidential data.

## Code availability

The underlying code for this study is not publicly available for proprietary reasons. Code for this study was written using Python (version 3.8) and R (version 4.2.2).
