# Supplementary material for: A Novel method for quantifying fluctuations in wearable derived daily cardiovascular parameters across the menstrual cycle
Source: NPJ Digit Med. 2024 Dec 23;7:373. doi: 10.1038/s41746-024-01394-0 (PMC11666598; doi:10.1038/s41746-024-01394-0)

Supplementary Note 1

In a supplementary analysis, identical unadjusted and adjusted GAMs as described in **Equations (1, 2)** were applied to individuals reporting using oral hormonal birth control. In the unadjusted model, the day of the menstrual cycle was a significant variable for both the RHR ($p<0.001$) and RMSSD ($p<0.001$) offsets. In the adjusted model, the day of the menstrual cycle (HRV $p<0.001$, RHR $p<0.001$), daily energy expenditure (KJ) (RMSSD $p<0.001$, RHR $p<0.001$), and weekend status (RMSSD $p<0.001$, RHR $p<0.001$) were significant. Age was not a significant factor in relation to RMSSD or RHR in this analysis (RMSSD $p=0.234$; RHR $p=0.061$) (**Supplementary Tables 1 & 2)**. The partial dependence plots of the day of the menstrual cycle for the offset from cycle mean in RHR and RMSSD are shown in **Supplementary Figure 1**. The pattern throughout the cycle, as shown in **Supplementary Figure 1**, illustrates an increase in RMSSD offset and a decrease in RHR offset during menses, followed by a regression to baseline following the conclusion of menses (approx. day 5).

Supplementary Figure 1. RHR and RMSSD offset across the menstrual cycle in naturally cycling individuals and individuals using hormonal birth control.

The partial dependence plot of the day of the menstrual cycle from the population-level GAMs for the cohort using hormonal birth control pills and the naturally cycling cohort. Naturally cycling individuals are represented with the black dashed line, participants using birth control pills are shown with a grey dotted line. Offset from the participant's mean RHR is shown in (a) and from the participant's mean RMSSD is shown in (b). A red line indicates the mean end of menstrual bleeding. For each variable, the central line provides the estimate, with the upper and lower lines providing the 95% confidence interval.

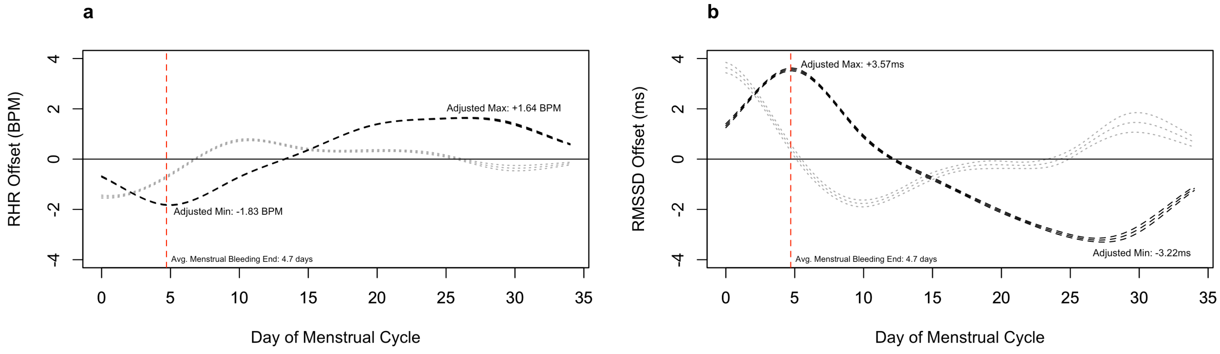

Supplementary Table 1: Model Statistics: RHR model, Birth Control

Estimates, Confidence Intervals (CI), test statistics, and p-values for the predictors in the adjusted (n=159,542) and unadjusted (n= 165,236) population GAM models of RHR offset. This model illustrates the relationship between D, pID, weekend, BMI, age, and kJ on the offset of RHR throughout the menstrual cycle in individuals taking Birth Control Pills.

	**Birth Control: Unadjusted RHR**				**Birth Control: Adjusted RHR**			
Predictors	*Estimates*	*CI*	*Statistic*	*p*	*Estimates*	*CI*	*Statistic*	*p*
Intercept	0.00	-0.02 – 0.02	0.00	0.997	-0.52	-0.63 – -0.42	-10.00	**<0.001**
D			1617.41	**<0.001**			1048.79	**<0.001**
pID			0.00	0.958			0.67	0.364
weekend (True)					0.96	0.92 – 0.99	49.03	**<0.001**
BMI					0.01	0.01 – 0.01	5.04	**<0.001**
age							3.52	0.061
kJ							313.12	**<0.001**
Observations	165236				159542			
R^2	0.038				0.059			

Supplementary Table 2: Model Statistics: RMSSD model, Birth Control

Estimates, Confidence Intervals (CI), test statistics, and p-values for the predictors in the adjusted (n=161,124) and unadjusted (n=167,024) population GAM models of RMSSD *offset. This model illustrates the relationship between D, pID, weekend, BMI, age, and kJ on the offset of* RMSSD *throughout the menstrual cycle in individuals taking Birth Control Pills.*

	**Birth Control: Unadjusted RMSSD**				**Birth Control: Adjusted RMSSD**			
Predictors	*Estimates*	*CI*	*Statistic*	*p*	*Estimates*	*CI*	*Statistic*	*p*
Intercept	-0.00	-0.06 – 0.06	-0.00	0.998	1.38	1.00 – 1.76	7.11	**<0.001**
D			506.71	**<0.001**			341.14	**<0.001**
pID			0.00	0.972			0.60	0.392
weekend (True)					-2.04	-2.18 – -1.90	-28.37	**<0.001**
BMI					-0.03	-0.05 – -0.02	-4.20	**<0.001**
age							1.66	0.234
kJ							258.54	**<0.001**
Observations	167024				161124			
R^2	0.012				0.023			

Supplementary Note 2

Subgroup Analysis of Age-Matched Birth Control Cohort

As the birth control pill cohort was composed of significantly younger females, an age matched subgroup analysis was carried out to compare more demographically similarly groups. After matching, the age matched subgroup for the naturally cycling cohort (n=1,661) had a mean age of 30.9 years (± 6.60) with a BMI of 24.55 kg/m^2 (±3.92), while the birth control bill cohort (n=1,661) had a mean age of 30.9 years (± 6.60) and BMI of 24.30 kg/m^2 (±4.10). For cardiovascular amplitudes, the mean value of RHR_{amp} is 2.84 bpm (±1.99) for those naturally cycling, and 0.28 bpm (±1.94) for participants using birth control. For $RMSSD_{amp}$, the mean value is 5.37 ms (±7.98) for those naturally cycling and 0.51 ms (±6.70) for participants using birth control pills. The mean values of both RHR_{amp} and $RMSSD_{amp}$ differ significantly (RHR_{amp}, $p<0.001$; $RMSSD_{amp}$, $p<0.001$) between the birth control and naturally cycling cohorts (**Supplementary Figure 2)**.

Supplementary Figure 2: Distribution of Novel Cardiovascular Amplitude Metric for Age-matched Subgroups of Participants Using a Birth Control Pill and Naturally Cycling Participants.

Histogram plots for the distributions of RHR_{amp} values are shown in (a) and the distributions of $RMSSD_{amp}$ values are shown in (b). In both (a) and (b), the age-matched naturally cycling cohort is displayed in light gray with the cohort mean represented by the (---) line, while the age-matched birth control pill cohort is displayed in dark gray with the cohort mean represented by the (...) line.

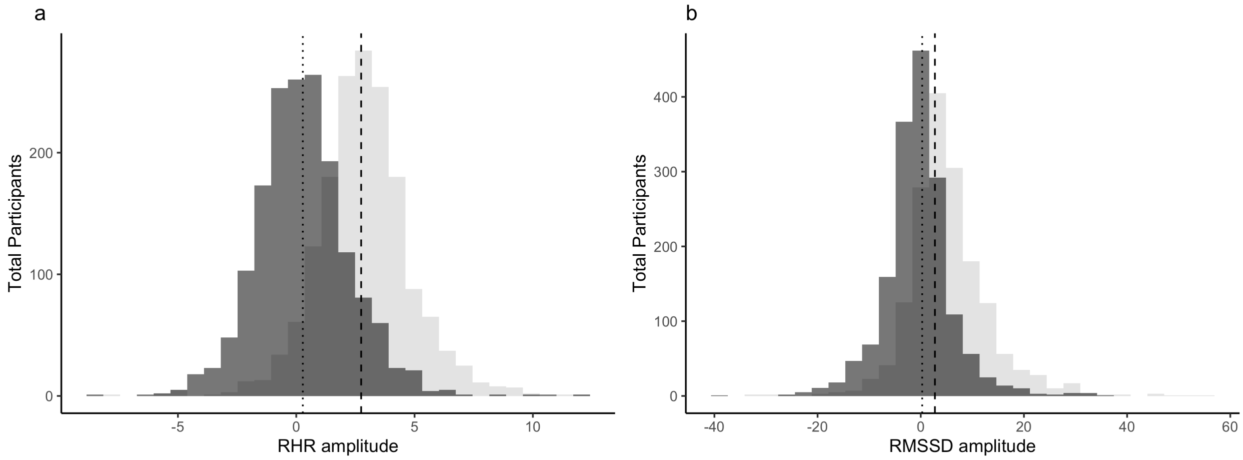

Supplement: Supplementary file 1 — Supplementary File [file 41746_2024_1394_MOESM1_ESM.pdf]
